# Prolactin receptor signaling: A novel target for cancer treatment - Exploring anti-PRLR signaling strategies

**DOI:** 10.3389/fendo.2022.1112987

**Published:** 2023-01-13

**Authors:** David Standing, Prasad Dandawate, Shrikant Anant

**Affiliations:** Department of Cancer Biology, University of Kansas Medical Center, Kansas City, KS, United States

**Keywords:** PrlR, antagonist, small molecule inhibitor, immunotherapy, antibody-drug conjugate

## Abstract

Prolactin (PRL) is a peptide hormone mainly secreted from the anterior pituitary gland. PRL is reported to play a role in pregnancy, mammary gland development, immune modulation, reproduction, and differentiation of islet cells. PRL binds to its receptor PRLR, which belongs to a superfamily of the class I cytokine receptor that has no intrinsic kinase activity. In canonical signaling, PRL binding to PRLR induces downstream signaling including JAK-STAT, AKT and MAPK pathways. This leads to increased cell proliferation, stemness, migration, apoptosis inhibition, and resistance to chemotherapy. PRL-signaling is upregulated in numerous hormone-dependent cancers including breast, prostate, ovarian, and endometrial cancer. However, more recently, the pathway has been reported to play a tumor-promoting role in other cancer types such as colon, pancreas, and hepatocellular cancers. Hence, the signaling pathway is an attractive target for drug development with blockade of the receptor being a potential therapeutic approach. Different strategies have been developed to target this receptor including modification of PRL peptides (Del1-9-G129R-hPRL, G129R-Prl), growth hormone receptor/prolactin receptor bispecific antibody antagonist, neutralizing antibody LFA102, an antibody-drug conjugate (ABBV-176) of the humanized antibody h16f (PR-1594804) and pyrrolobenzodiazepine dimer, a bispecific antibody targeting both PRLR and CD3, an *in vivo* half-life extended fusion protein containing PRLR antagonist PrlRA and albumin binding domain. There have also been attempts to discover and develop small molecular inhibitors targeting PRLR. Recently, using structure-based virtual screening, we identified a few antipsychotic drugs including penfluridol as a molecule that inhibits PRL-signaling to inhibit PDAC tumor progression. In this review, we will summarize the recent advances in the biology of this receptor in cancer and give an account of PRLR antagonist development for the treatment of cancer.

## 1 Introduction

**Figure 1:**
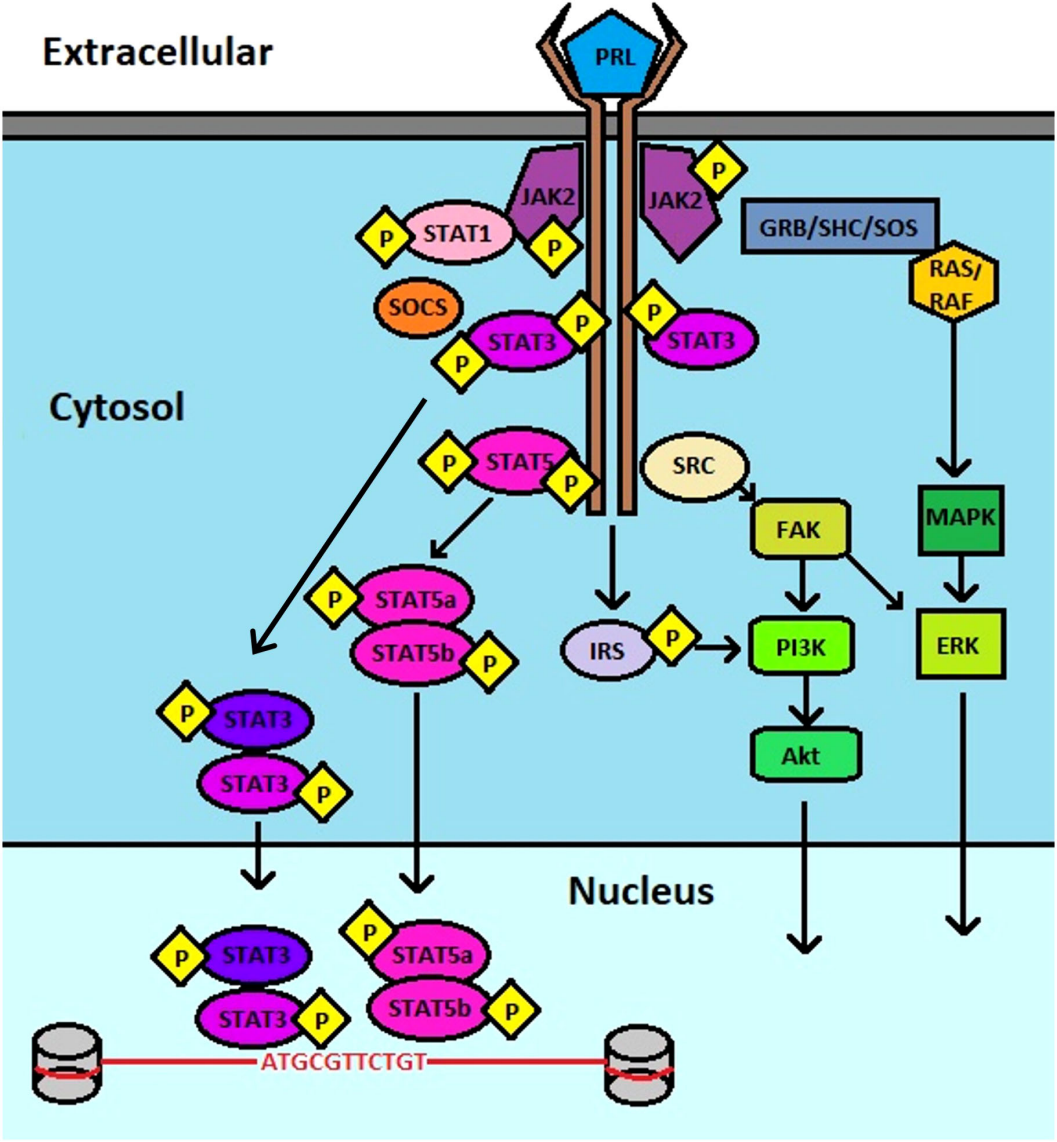
Schematic of PRL : PRLR signaling. PRL binds to PRLR, inducing JAK2 association that leads to downstream activation of multiple pathways that include STAT3, STAT5, PI3K, AKT, and ERK.

Prolactin (PRL) and its cognate receptor, prolactin receptor (PRLR), have been characterized in hundreds of biological functions, especially mammary gland development and lactation. PRL is a peptide hormone that resembles the growth hormone due to a conserved helix bundle composition. It is largely produced by the lactotrope cells of the anterior pituitary gland as a pro-hormone that undergoes proteolytic cleavage to produce a 199 amino acid active peptide (1). However, aberrant PRL levels are also observed in disease states, which may also be related to its synthesis from the affected tissues including the prostate, skin, adipose tissue, endometrium, myometrium, immune cells, brain, and breast tissues (2). It can therefore participate in paracrine and autocrine signaling functions related to cell homeostasis and growth (3). Composed of 4 parallel alpha helices, PRL, binds to PRLR *via* several residues, including Lys-69, Tyr-169, and H180 of Site 1, and Arg-24, Lys-124 within the Gly129 cavity and Glu-43 within the N-terminus of Site 2, stimulating dimerization of PRLR on the cell surface, leading to activation of canonical signaling *via* Janus kinase (JAK)-signal transducer and activator of transcription (STAT) (**Figure 1**) (4–8).

Extrapituitary prolactin is thought to be regulated primarily at the transcriptional and translational level. In contrast, lactotrope cells have large vacuolar stores of PRL, which can be released by calcium-dependent exocytosis. Transcription of PRL mRNA in tissues other than the pituitary is regulated by an alternative promoter upstream of the site utilized by lactotrope cells (9). Transcripts generated from alternative promotor driven transcription results in inclusion of an additional exon1a within the 5’untranslated region of the transcript. However, this does not alter the amino acids of the encoded protein (10). While pituitary PRL synthesis and release is sensitive to regulation by dopamine, typically extrapituitary PRL is not (11). An exception to this is in the context of adipocytes in which PRL is dependent on dopamine (12). The mechanisms that control expression of PRL at extrapituitary sites is poorly understood; however, the use of an alternate promoter indicates site specific regulation of PRL transcription to modulate expression, which warrants further study especially during tumorigenesis (13).

PRLR is a type 1 cytokine receptor, encoded by the PRLR gene on chromosome 5. Conserved homology permits binding by human growth hormone (GH) in addition to PRL. In humans, the PRLR gene contains 11 exons and is widely expressed throughout the body (14). PRLR can undergo alternatively splicing events resulting in the expression of several PRLR isoforms, with tissue specificity. These isoforms have modified cytoplasmic domains, but share identical extracellular domains that bind PRL. Moreover, PRLR lacks intrinsic kinase activity, thus necessitating dependency on associated kinases such as the Janus kinases (JAKs) to further transduce signaling. PRLR is a single pass transmembrane protein that has two conserved cytoplasmic regions, Box1 and Box2, which are responsible for association with JAK2 (15).

PRLR signaling plays a major role in numerous biological functions, primarily mammary gland development and lactation. However, due to the widespread expression of PRLR within tissues, aberrant activation of this signaling has been linked to progression of prostate, breast, cervical, ovarian, and pancreatic tumors (16).

High expression of PRLR and circulating PRL can drive the expression of genes involved in proliferation, migration, and invasion of cancer cells. In breast cancer, PRL-mediated JAK/STAT signaling contributes to endocrine therapy resistance in conjunction with elevated HER2, by activating oncogenic factors such as MYC, FOS, and JUN (17). This has been shown to be mediated, in part, by the estrogen independent activation of ERα by PRL, both *in vitro* and *in vivo* (18, 19). In particular, PRL has been shown to activate ERα through a PAK1 mediated mechanism, circumventing the mechanism of action of anti-estrogen therapies (20). Others have shown that PRL participates in endocrine therapy resistance through the activation of PRLR, and stimulating downstream signaling pathways that include STAT5, ERK1/2, and PI3K (20–22). With prostate cancer, PRL overexpression contributes to increased hyperplasia of prostatic tissues, thereby elevating the risk for developing adenocarcinomas. Epidemiologic studies have linked PRL and STAT5 with higher grade tumors and more aggressive disease (23). Enhanced PRLR signaling in gynecological, pancreatic, and colorectal tumors promotes metastatic potential, chemoresistance, and pro-survival signaling events (24–27). Briefly, preincubation of PRL for 1 hour abrogated cisplatin-induced apoptosis of ovarian and endometrial cancer cells, as determined by Annexin V/PI staining (27). The authors demonstrate significant activation of Ras signaling, as well as STAT3, ATF-2, MEK1, CREB, and p53 within 5 minutes of PRL stimulation (27). Interestingly, GH has been shown to induce the expression of ABC efflux transporters (ABCB1, ABCB5, ABCC1, ABCC2, ABCG1, and ABCG2), contributing to acquired drug resistance (28). Concurrently, PRL has been shown to induce the expression of ABCG2 through the activation of STAT5, leading to binding at consensus sequences upstream of the ABCG2 transcription start site (29). Moreover, the authors further demonstrated that STAT5 was required, but insufficient for PRL induced transcription, as MAPK and PI3K inhibitors also decreased PRL induced ABCG2 expression, without affecting STAT5 DNA binding (29). In our own studies with pancreatic cancer, we observed that PRLR signaling potentiated invasive cell behavior and stemness through JAK2/STAT3 and ERK phosphorylation (25). We had previously observed in colon cancer, that PRL enhanced stemness in a JAK2/STAT3/ERK dependent manner by modulating Notch signaling (26). Interestingly, in both pancreatic and colon cancer, we did not observe activation of STAT5 (25) As such, PRLR signaling plays an extensive role in human cancers, which has led to research directed towards developing therapeutic strategies to modulate activity.

Due to the strong evidence supporting the critical role of PRL and PRLR in human cancers, various approaches have attempted to modulate activity both by suppressing downstream signaling as well as by developing PRLR antagonists. These strategies will be discussed in more detail later. In brief, the use of a PRL antagonist peptide, G129R, was shown to block the PRL : PRLR signaling axis in ovarian cancer mouse models (30). This resulted in greater than 90% reduction in tumor weights compared to controls, when used in combination with the standard-of-care agent paclitaxel. A preclinical study of the anti-PRLR antibody REGN2878-DM1 suggested induction of cell-cycle arrest and apoptosis in PRLR expressing breast cancer cell lines, and also exhibited synergistic activity with fulvestrant (31). In preclinical studies with pancreatic cancer, we identified a small molecule Penfluridol to inhibit PRL induced JAK/STAT activation by competitively binding to PRLR. This resulted in suppression of cancer cell growth *in vitro* and *in vivo* (25). The efficacy of these preclinical studies demonstrates the validity of targeting PRLR while also establishing the critical role of PRL : PRLR signaling in human cancers.

In this review, we discuss the current research strategies directed towards PRL : PRLR inhibition. Due to the extensive expression of PRL and PRLR in various tissues, and the efficacy of preclinical inhibitory strategies, it is clear that the PRL : PRLR signaling axis is a critical pathway in human biology and cancers.

## 2 Novel approaches to target prolactin receptor

### 2.1 Competitive antagonists of the human prolactin

A class of inhibitors that was first developed to target prolactin-sensitive pathologies such as dopamine-resistant prolactinomas, as well as breast, prostate and pancreatic malignancies were designed to compete with endogenous PRL for PRLR binding (32). As such, these types of antagonists often require higher molar concentrations compared to endogenous PRL to ensure sufficient activity (33). Moreover, it is vital that any unintended agonistic properties are eliminated, particularly at high concentrations (33). As described previously, PRLR signaling is activated by the binding of PRL to a PRLR homodimer. This interaction is ternary in nature and has 3 intermolecular interactions referred to as sites 1-3. Site 1 and 2 interactions are between prolactin and each receptor, while site 3 is the interaction between two receptor units. Once active, this ternary complex induces various downstream signaling pathways, including the JAK2-STAT3/STAT5 axis, MAP kinase, AKT, and Src kinase pathways (8, 34). It is this ternary interaction between PRL and PRLR that has served as the design template for the development of competitive PRLR antagonists, such as G129R-hPRL and Del1–9-G129R-hPRL, which will be discussed in detail below.

#### 2.1.1 G129R-hPRL

G129R-hPRL was developed in the early 1990s as the result of a mutational screen of hPRL with the purpose of identifying and characterizing binding sites in PRLR. This was based on strategies that were utilized for growth hormone (GH) and its cognate receptor (GHR) that yielded the discovery of a potent GHR antagonist and drug, Pegvisomant (7, 35–39). G129R-hPRL was tested for its inhibitory activity in the NB2 rat cell proliferation assay, because PRL induces proliferation of these cells. Surprisingly, instead of being an antagonist, G129R-hPRL appeared to actually be a weak agonist, increasing the proliferation of NB2 cells rather than suppress it (7). Binding of G129-hPRL was confirmed by surface plasmon resonance; however, the affinity towards site 2 of PRLR was demonstrated to be decreased compared to WT hPRL (6, 7, 40). Based on these findings, it was concluded that the lack of antagonistic activity was due, at least in part, to poor affinity for site 2, leading to insufficient hindrance of ligand:receptor interaction. Shortly after these initial reports, several studies determined that detection of the competitive antagonistic properties of G129-hPRL was impacted by the bioassay used and species of origin (41–43). A PRL-responsive luciferase reporter assay was designed in human embryonic kidney fibroblasts (HEK293) that were transfected with a hPRLR long isoform expressing construct. Under these conditions, G129R-hPRL exerted potent antagonistic activity (6). Species specific discrepancies were also confirmed, as G129R-hPRL had reduced antagonism towards rat PRLR (6). These findings were validated by multiple groups using various hPRLR-mediated cell bioassays and breast cancer cell lines (44–47). However, conflicting results were obtained when studies were performed in Ba/F03 human cells stably transduced with hPRLR (Ba/F03-hPRLR). When stimulated with hPRL, Ba/F03-hPRLR cells exhibited increased proliferation, while G129R-hPRL failed to induce antagonistic effects, similar to results obtained previously in NB2 rat cells (48). As such, it was hypothesized that G129R-hPRL behaves as a weak antagonist/partial agonist in sensitive bioassays, while in low sensitivity assays where the levels of PRLR activation induced by G129R-hPRL is not sufficient to produce a biological effect, it acts as an antagonist (48). Despite these contradictory findings, many studies have since been performed demonstrating antagonistic activity, which are outlined below.

In a recent report, it has been shown that G129R-hPRL blocks the activity of PRL-PRLR signaling in ovarian cancer (30). The authors demonstrate that in orthotopic mouse models G129R-hPRL inhibits tumor growth in a dose-dependent manner. Moreover, prolonged treatment with G129R-hPRL at 100 μg/day resulted in a durable response, and reduced tumor weights by 50% compared to control, while in combination with paclitaxel produced more than a 90% inhibition (30). There was also no apparent off target toxicity with G129R-hPRL. In *in vitro* studies, the authors further demonstrated that G129R-hPRL did not inhibit proliferation or migration in 2-dimensional monolayer cultures of SKOV3 cells; however, in 3-dimensional spheroid cultures of HeyA8 and SKOV3 cells, G129R-hPRL abrogated cellular growth and induced apoptosis (30). Furthermore, G129R-hPRL attenuated PRL induced growth and activation of JAK2, STAT3, and STAT5 phosphorylation in HeyA8 cells, further supporting the antagonistic properties of G129R-hPRL (30).

Several groups have studied the role of PRLR in breast cancer, and in the process have observed antagonistic activity in cells treated with the G129R-hPRL analog. Chen et al. showed that G129R-hPRL treatment inhibited proliferation of T47D breast cancer cells and induced apoptosis within 2 hours of treatment at a dose of 50 ng/mL (45). In regard to PRLR signaling, Catalado et al. sought to determine the effect of G129R-hPRL on STAT3 activation, and identified that hPRL activated STAT3 preferentially compared to STAT5 in T47D breast cancer cells (47). Furthermore, the authors determined that G129R-hPRL inhibited STAT3 phosphorylation (47). This was further confirmed by others, in which G129R-hPRL attenuated PRL-induced activation of JAK-STAT and MAPK pathways (44). In breast cancer xenograft models, PRL was found to induce tumor growth of T47D and MCF-7 tumors, while G129R-hPRL inhibited growth (49). These findings provide evidence that targeting the PRL : PRLR signaling axis is feasible and that the G129-hPRL has antagonistic activity. As a result, further interest in targeting PRLR has led to several studies focused on developing G129R-hPRL fusion proteins as well as combinatorial therapeutic strategies.

A fusion protein of G129R-hPRL with Pseudomonas exotoxin A (PE40) was developed and found to competitively bind to hPRLR in T47D cells, further suppressing PRL-induced STAT5 phosphorylation and inducing caspase-independent cytotoxicity (50). In another study, G129R-hPRL was fused to endostatin, and was shown to inhibit PRL-induced signaling in T47D breast cancer cells, while further suppressing HUVEC cell proliferation, tube formation, and tumor formation of mouse 4T1 cells *in vivo* (51, 52). Tomblyn et al. have examined the combination of three G129R-hPRL based fusion proteins, which include G129R-hPRL fusions with endostatin (an angiogenesis inhibitor), interleukin 2 (immune modulator), and PE38KDEL (a truncated cytotoxin) in allografts of a mammary carcinoma cell line (McNeuA) derived from MMTC-neu mice (53). Treatment with these fusion proteins increased the number of cytotoxic CD8+ T cells in the tumor, while reducing recurrence and lung metastases (53). In similar studies conducted by Scotti et al, combining G129R-hPRL with Herceptin resulted in suppression of STAT3 and STAT5 phosphorylation and reduced HER2 expression in T47D and BT474 breast cancer cells (54). The combination of G129R-hPRL with Herceptin also demonstrated an additive inhibitory effect on HER2 and MAPK activation and further suppressed tumor xenograft growth in athymic nude mice (54). Taken together, these studies demonstrate antagonistic activity of G129R-hPRL, despite previous confounding studies, and show the feasibility of inhibiting PRLR signaling to suppress cancer growth.

#### 2.1.2 Δ1–9-G129R-hPRL

Due to confounding evidence of agonistic activity of G129R-hPRL, development of a second-generation PRLR antagonist was attempted, resulting in a competitive antagonist that is devoid of residual agonistic properties in cell culture and animal models (55). The Δ1–9-G129R-hPRL is a human prolactin core protein analog that has two modifications: 1) a deletion of nine N-terminal amino acid residues and 2) a glycine substitution by arginine at residue 129 (55). This second-generation PRLR antagonist was developed following findings from G129R-hPRL. Furthermore, crystal structures of ovine placental lactogen (PL), a polypeptide that shares high structural and functional similarities with PRL, and rat PRL binding protein (PRLBP) identified that the N-terminal region of PL is critical in site 2 binding of PRLR (56). This finding led to in depth analyses of the N-terminal domain in PRL biological activity (57). Multiple deletion constructs were developed including deletion of amino acids 1-9 (Δ1-9-hPRL) and 1-14 (Δ1-14-hPRL) (58). Interestingly, the Δ1-9-hPRL construct increased receptor binding affinity and biological activity, while the Δ1-14-hPRL construct decreased binding affinity and activity by modulating site 2 functionality (55, 58). Although the effects were modest, these deletion mutations were introduced into G129R-hPRL, intending to improve upon the antagonistic properties of the parent construct. Both of the double mutant analogs, Δ1-9-G129R-hPRL and Δ1-14-G129R-hPRL, exhibited similar dose-response curves in bioassays of PRLR activity. These new analogs failed to improve upon the antagonistic properties of the first-generation construct, G129R-hPRL; however, the authors did observe significant improvements related to agonistic activity. While G129R-hPRL displayed agonistic properties in sensitive bioassays, particularly Ba/F-LP and Nb2 cell proliferations assays, the new double mutant analogs failed to stimulate proliferation. These data demonstrate that the absence of agonistic activity markedly improved the second-generation antagonists.

Goffin et al. published a crystallographic structure of Δ1-9-G129R-hPRL to understand the structural and thermodynamic basis of PRLR antagonism (56). The authors reported no major structural changes compared to wild type hPRL, suggesting the pure antagonistic properties of Δ1-9-G129R-hPRL are due to intrinsic mutations and deletions (56). Moreover, they compared the physiochemical, structural, and biological properties of wild type hPRL and various variants including N-terminal or Gly129 mutations, either alone or in combination. The authors determined that human PRL activity was unaffected by N-terminal modifications; however, in the context of G129R mutants, N-terminal deletions eliminated residual agonist activity. Moreover, this was unrelated to site 1 affinity (56). Conversely, N-terminal alterations impacted biological activity only when site 2 binding was affected by G129 mutants (56). N-terminal deletions of PRL did have measurable decreases in site 2 affinity alone, as determined by SPR; however, these modifications were insufficient to eliminate biological activity indicating the critical nature of G129 to hPRL function (56). What this indicates is twofold: 1) that the N-terminus participated in site 2 binding and 2) that residual agonism of early PRL antagonists may be eliminated by further modifying the N-terminus interactions with site 2.

Several studies have employed the second-generation antagonist to dissect PRL : PRLR biology. Ferraris et al. studied the effects of Δ1-9-G129R-hPRL in the turnover of mouse anterior pituitary cells and PRLR expression *in vivo* using transgenic mice constitutively expressing the analog (59). The authors observed that the weight and proliferation index of the pituitary gland was elevated in transgenic mice expressing the antagonist compared to wild type mice (59). Moreover, *in vitro* studies showed that Δ1-9-G129R-hPRL enhanced proliferation while reducing apoptosis of GH3 cells, a somatolactotrope and primary rat anterior pituitary cells (59). These data suggest that PRL acts as an antiproliferative and pro-apoptotic factor in cells of the anterior pituitary gland. Dwivedi et al. identified hematopoietic PBX-interacting protein (HPIP) as a novel regulator of mammary epithelial cell differentiation, where Δ1-9-G129R-hPRL attenuated HPIP-mediated synthesis of PRL, activation of AKT, and synthesis of β-casein in cultured HC11 cells (60). Recently, synthesis and purification of Δ1-9-G129R-hPRL was performed by testing different activation temperature and chromatographic techniques including nickel-affinity chromatography, size-exclusion chromatography and high-performance size-exclusion chromatography (HPSEC) (61). Δ1-9-G129R-hPRL was extracted with more than 95% purity, enhanced solubility, correct folding, and without methionine, and has a significant potential in clinical application (61).

In the context of cancer, several groups have shown anti-tumor activity and suppression of PRLR signaling following treatment with the Δ1-9-G129R-hPRL antagonist. Treatment with Δ1-9-G129R-hPRL abolished the increase in nitric oxide production by prolactin-induced plasma membrane carboxypeptidase D in triple-negative breast cancer cell lines (62). It was further shown to inhibit prolactin-induced osteoclast differentiation and bone lysis in breast cancer cells (63). Similar inhibition of PRL-induced carboxypeptidase D was also seen in prostate cancer (64). In addition, Hou et al. demonstrated that while PRL increased oncogenic potential in breast cancer cells by stimulating HOXA1, which in turn induced STAT5, ERK phosphorylation, and increased transcriptional activity of ELK1, SAP1A, STAT5A and B to increase cell proliferation, survival and anchorage dependent growth, following treatment with Δ1-9-G129R-hPRL (65). The effect of Δ1-9-G129R-hPRL induced PRLR antagonism was further studied by Howell et al. in multiple breast cancer cell lines (66). As a monotherapy Δ1-9-G129R-hPRL failed to demonstrate antiproliferative effects of the cell lines, but potentiated the effects of doxorubicin and paclitaxel when used in combination (66). Moreover, Δ1-9-G129R-hPRL inhibited the growth of colonies in soft agar and mammosphere formation supporting the rational for use in combination therapeutic strategies for breast cancer (66). Asad et al. have studied the effects of PRLR inhibition on glioblastoma multiforme (GBM) pathogenesis (67). The authors identified that PRLR was highly expressed and was further correlated with poor survival in GBM patients (67). Moreover, Δ1-9-G129R-hPRL treatment reduced the proliferation, colony formation, chemoresistance and migration in GBM cells suggesting potential for PRLR as a therapeutic target in GBM (67). Lastly, Δ1-9-G129R-hPRL treatment prevented early stages of prostate carcinogenesis by inhibiting STAT5 phosphorylation, proliferation, abnormal basal-cell pattern and grade of intraepithelial prostate neoplasia suggesting the application of PRLR-based therapies in prostate cancer (68). Collectively, these studies demonstrate antagonistic activity of Δ1-9-G129R-hPRL and further provide solid evidence for targeting PRLR in human malignancies.

#### 2.1.3 Improving half-life of PRLR antagonists *In vivo*

While current PRLR antagonists have shown promise in pre-clinical applications, there remain challenges limiting their usage in clinic. PRL and current PRL antagonists have molecular weights of ~23 kDa, which are below the 60kDa cut-off values for glomerular filtration by the kidneys (69). Hence, these are quickly cleared from the blood following intravenous delivery. Hence, the half-life of PRL in the blood is ~41 minutes (70), and speculation towards PRLR antagonists would yield similar results. As such, their application in a clinical setting is limited.

To overcome this challenge, Yu et al. have developed a PRLR antagonist fusion protein designed around Δ1-9-G129R-hPRL, and several additional mutations (C11S, S33A, Q73L, G129R and K190R). In addition, the fusion protein included an albumin binding domain (ABD) from Streptococcal protein G, also known as ABD_035_, which has 46 amino acids in a three-helix structure (71). Surface plasmon resonance of this fusion protein, called PrlRA-ABD determined the K_D_ to be 2.3 ± 0.2 vs 3.4 ± 0.5 nM of PrlRA alone, while PRL showed a KD value of 23 ± 4 nM (71). Furthermore, ABD-PrlRA and PrlRA both inhibited PRL-induced phosphorylation of STAT5 in U251-MG cells in a dose-dependent manner (71). To understand the changes in pharmacokinetics both PrlRA and ABD-PrlRA were injected subcutaneously in Wistar rats at a dose of 4 mg/kg. After 24 h, serum was analyzed for PrlRA and ABD-PrlRA concentration and determined to be 150 ng/ml and 15,000 ng/ml, respectively (71). This data suggests that addition of ABD to PrlRA enhanced its *in vivo* half-life by 100-fold, demonstrating the feasibility of *in vivo* applications.

Additional strategies that have been effective for hGH may also have implications for PRL antagonists. Pegvisomant is a PEGylated G120K protein analog of hGH, and was the first drug approved as a GHR antagonist (39). Much like PRL, hGH is readily cleared by kidney filtration. To slow clearance, polyethylene glycol (PEG) polymers were attached to hGH derivatives. The authors observed significant retention of PEG-hGH derivatives in serum compared to hGH, with measurable concentrations detected out to ~200 hours and 12 hours, respectively (37). Since PEGylation of hGH derivatives proved successful, we could speculate that these strategies may be useful for PRL antagonists as well; however there are challenges that must be overcome with the use of PEG based polymers. PEG chains can mask the protein binding sites, and thereby reduce affinity of biological activity (72). Therefore, design of the polymer is crucial to developing an effective PEGylated protein. Nevertheless, this may provide additional opportunities for improving PRL antagonist half-lives, and warrant further study.

### 2.2 Antibody-based PRLR antagonists

The use of antibody-based therapeutic agents has become attractive and one of the most successful strategies for the treatment of various diseases, including cancer (73). The use of monoclonal antibodies has achieved significant success in recent years, while antibody-drug conjugates have only recently been utilized for the treatment of solid tumors and lymphomas (73). The anticancer effects of these monoclonal antibodies can be due to direct receptor blockade, immune-activated cell killing, and specific defects of antibodies on cancer vasculature and stromal components as well as drug delivery (74–77). Specific examples of successful monoclonal antibody therapies targeted epidermal growth factor receptor (EGFR) (75, 78), C-MET (79), HER2 (80), fibroblast activation protein (FAP) (81), and cytotoxic T lymphocyte-associated antigen 4 (CTLA4) (82). The ideal properties of monoclonal antibodies include high selectivity towards specific target antigens, activating immune cell responses, and modulating downstream signaling pathways (83). Hence, antibody design is critical for successful preclinical and clinical applications. The successful development of monoclonal antibodies for use in a clinical setting involves identification of the physiochemical properties of the antibody, analysis of specificity, study of immune response and signaling pathways as well as *in vivo* antibody localization, biodistribution, toxicity, and efficacy (73). Several monoclonal antibodies have received approval by US Food and Drug Administration in the recent decade, which have been summarized previously (84–87). In the context of PRLR, the presence of a defined extracellular domain structure makes it an attractive target for designing monoclonal antibody based inhibitors and therapeutics. As such, several antibodies and antibody based constructs have been developed targeting PRLR and are being tested in preclinical and clinical studies, which will be summarized in detail in the following sections.

#### 2.2.1 PRLR neutralizing antibodies

##### 2.2.1.1 LFA102

Damiano et al. developed and characterized a neutralizing antibody LF102 targeting human PRLR, which was shown to inhibit the physiological functions of both autocrine and paracrine PRL (88). The authors first generated a parental hybridoma to LFA102 in mice immunized with recombinant PRLR extracellular domain, then LFA102 was prepared by humanizing the antibody (88). Using flow activated cell sorting, the authors demonstrated that LFA102 binds to PRLR in human breast cancer cell lines, in addition to primary breast cancer cells (88). Moreover, LFA102 was also found to bind to rat pre-T cell lymphoma cell line Nb2-11 suggesting this antibody has cross-reactivity to rat PRLR (88). To assess selectivity of LFA102, the authors used a PRLR-negative BaF3 cell line and re-expressed PRLR (BAF3-PRLR). LFA102 did not bind to PRLR-negative BaF3 cells but was found to bind to BaF3-PRLR (88). In addition, the antibody did not interact with cells expressing murine PRLR. To determine if LFA102 acted through a competitive or non-competitive mechanism with PRLR, the authors designed a ligand competition assay using Alexa647-labeled PRL (A647-PRL). The authors demonstrated that LFA102 did not affect A647-PRL binding to PRLR even at saturation concentrations of LFA102 (88), suggesting that LFA102 is not a ligand-competitive inhibitor. To determine whether LF102 affects PRL-mediated signaling, T47D breast cancer cells were treated with the antibody. There was significant attenuation of PRL-induced phosphorylation of STAT5, AKT, and ERK in a concentration-dependent manner (88). However, LFA102 failed to regulate PRLR signaling when treated alone demonstrating the absence of residual agonistic activity.

As proof of principle for *in vivo* activity, T47D-T2 xenografts were generated in NOD**/**SCID mice and LFA102 or as control, a human IgG1 was administered, followed by a bolus of PRL to stimulate PRLR. Mice treated with PRL alone showed increased levels of phosphorylated STAT5 in the tumors, while the treatment of LFA102 inhibited this PRL-induced phosphorylation suggesting the *in vivo* efficacy of LFA102 (88). In addition, LFA102 achieved a 30-56 μg/mL concentration in the serum of these mice. Detailed pharmacokinetic and pharmacodynamics studies of LF102 were subsequently performed. The clearance of LFA102 ranged from 1.45 to 0.92 mL/h/kg and 13.5 to 3.93 mL/h/kg in males and females, respectively. In addition, the mean estimated half-life was between 1.43 and 8.99 days and 0.12 to 4.23 days in males and females, respectively (88). Subsequently, to further understand the antitumor efficacy of LF102, a subcutaneous xenograft model in SCID mice was used. Mice were injected subcutaneously with luciferase expressing Nb2-11 cells to generate tumors. These mice were treated with a single dose of LFA102 (0.01-10 mg/kg) or control IgG antibody, and disease burden was measured from 14 days after dosing to 4.5 months. Doses exceeding 0.3 mg/kg displayed antitumor efficacy by day 3 post-injection. Moreover, LFA102 treated mice (doses exceeding more than 0.3 mg/kg) showed significantly higher survival compared to controls, with 50% of animals surviving 2-4 fold longer than IgG1 treated mice (88).

In addition to xenograft tumor models, a carcinogen induced model was utilized to assess LFA102 efficacy. Briefly, 7,12-Dimethylbenz[α]anthracene (DMBA) was administered to induce rat mammary tumors. LFA102 treatment (300 mg/kg) significantly reduced PRLR signaling and tumor growth in this rat mammary cancer model as a monotherapy and combination with letrozole (aromatase inhibitor, 10 μg/kg). LFA102 treatment reduced tumor volume to 809±279 mm^3^ from 1964±243 mm^3^ in case of control, while combination of LFA102 and Letrozole further reduced tumor volume to 436±144 mm^3^, suggesting synergistic/additive anticancer activity (88). These data demonstrated that LFA102 has the potential to be the first effective antibody-based therapeutic agent for the treatment of PRL-responsive malignancies.

Following these studies, the clinical efficacy of LFA102 was assessed in patients with stage IV breast and castration-resistant prostate cancer. In this Phase I clinical trial, patients (n=73, female=34, male=39) received 3-60 mg/kg of LFA102 intravenously once every 4 weeks and the maximum tolerated dose (MTD) and/or recommended dose for expansion was determined to study the safety and antitumor efficacy of LFA102 (89). Drug-related toxicity was not observed during the dose escalation study, hence a MTD was not obtained during this study. The highest tested dose of 60 mg/kg was established as the recommended dose for expansion. The most common side effects recorded were fatigue (44%), nausea (33%), vomiting, constipation, and reduced appetite (21%), while 3 patients had adverse effects, which included decreased blood phosphorus, increased levels of serum lipase, and reduced blood lymphocyte count (89). The mean half-life of LFA102 ranged from 6-9 days. At the dose of 60 mg/kg, the C_max_ of LFA102 was found to be 1,495 ± 589 µg/ml, and mean area under the curve (AUC_last_) was 230,991 ± 102,673 hour × µg/ml (89). There was no response noted in the patients with breast cancer after LFA102 treatment. Similarly, in prostate cancer patients, there was no PSA response. Overall, LFA102 treatment contributed to stable disease in 13 patients out of 73 (18%), while all other patients 67 out of 73 (92%) discontinued the study due to the cancer progression (89). This poor response of LFA102 was thought to be because of insufficient exposure. The authors retrospectively hypothesized that a more frequent dosing of LFA102, such as once every 2 weeks, would have resulted in durable PRLR inhibition and superior antitumor efficacy

In another Phase 1 Trial study in East Asian patients of Japanese ancestry with breast (n=7) and prostate (n=7), similar results were obtained for MTD and anti-tumor activity of LFA102. Here, the antibody was administered at a dose of 3-40 mg/kg intravenously every 4 weeks (90). There were 14 patients enrolled in the study and grade 1 or 2 toxicities were reported in 9 patients out of 14 (64%), while the most frequent toxicity reported was nausea in 3 patients (21%) (90). The mean AUC_last_ of LFA102 (40 mg/kg) was found to be 5674 ± 507 µg/ml×day, C_max_ was found to be 1089 ± 227 µg/ml, while median t_1/2_ was found to be 12.1 days (90). As with the previous Phase I trial, LFA102 did not display antitumor activity.

##### 2.2.1.2 Anti-prolactin receptor (PRLR) antibody, F56

Cui et al. sought to design a new PRLR antagonist using a hybridoma technique to develop a series of monoclonal antibodies (91). After screening these antibodies, F56 was selected that specifically antagonized PRLR as assessed by enzyme-linked immunosorbent assay (ELISA) and western blot. The authors performed epitome mapping which identified a common binding epitope between F56 and PRL. In subsequent experiments, the authors determined that F56 inhibited PRL binding to PRLR in a dose-dependent manner suggesting that the F56 epitope overlapped with the PRL-binding site. Furthermore, F56 treatment (0.1-5 μg/ml) inhibited PRL-induced STAT3/5, AKT, and ERK phosphorylation in CHO cells expressing PRLR and Nb2 cells in a dose-dependent manner, confirming the antagonistic activity of F56 (91). Moreover, F56 inhibited PRL-induced proliferation of Nb2 cells, corroborating molecular data. These preclinical studies identified F56 as the first PRLR antagonist that has an overlapping epitope as PRL, which has potential to treat PRL-dependent diseases. However, early phase clinical trials will be required to assess toxicity, and preliminary efficacy.

#### 2.2.2 Antibody-drug conjugates

##### 2.2.2.1 ABBV-176

Antibody-drug conjugates (ADCs) have become a popular therapeutic design concept that combines the specificity of antibodies and potency of payload/cytotoxic drugs. Currently, 5 antibody-drug conjugates have been approved for the treatment of four hematological malignancies and one for solid tumors. For the purpose of targeting PRLR, Anderson et al. designed a novel pyrrolobenzodiazepine antibody-drug conjugate, ABBV-176 (92). To generate the PRLR-specific antibody used to produce the ABBV-176 ABC, a standard hybridoma technique following immunization with the PRLR extracellular domain was employed. The lead antibody selected was h16f (PR-1594804) based on affinity, epitope binding and activity (92). Initial screening was performed on antibodies conjugated to monomethyl-auristatin payload and studied based on ability to inhibit proliferation of the BT474 cell line. Based on this, the lead candidate ABBV-1776 was selected from the panel for further analysis. Surface plasmon resonance was performed with ABBV-176 and the extracellular domain of human PRLR, showing a strong affinity with a K_D_ value of 1 nM (92). In bioassays of anti-tumor activity, ABBV-176 was found to inhibit the growth of various cancer cell lines including breast cancer (IC50 value = 0.0055-0.77 nM), prostate cancer (IC50 value = 0.01 nM), endometrial cancer (IC50 value = 0.6 nM), ovarian cancer (IC50 value = 0.16 nM), colorectal cancer (IC50 value = 0.11 nM) and liver cancer (IC50 value = 5.2-8.6 nM) (92). These IC50 values were highly dependent on PRLR expression (i.e. more PRLR receptors was associated with higher IC50 for ABBV-176). Moreover, ABBV-176 was found to be nontoxic to normal/immortalized cell lines in kidney, breast, liver, lung, prostate, and vascular endothelium. Furthermore, the antitumor activity of ABBV-176 was evaluated in the BT-474 FP2 human xenograft breast cancer model. The single dose of 0.5 mg/kg was effective in significantly reducing tumor growth (92). A higher dose of 3 mg/kg produced the highest tumor reduction without affecting body weight as compared to control. Furthermore, there were no apparent physiological changes suggesting no impact on normal tissues. Similar results were obtained in a patient-derived xenograft (PDX) model. In these studies, the authors established the effect of ABBV-176 (0.1 mg/kg) in combination with the PARP inhibitor Valiparib (200 mg/kg) in CTG-0670 triple-negative, BRCA1 deficient, BRCA2 mutant PDX tumor models (92). It was determined that ABBV-176, both as a monotherapy and in combination with Valiparib, significantly inhibited PDX growth. This data suggests that ABBV-176 may be an effective therapy either alone or in combination with PARP inhibitors for the treatment of breast cancers (92).

Recently, Lemech et al. conducted a first-in-human Phase 1 dose-escalation study of ABBV-176 in patients with advanced solid tumors for evaluating safety, pharmacokinetics, and preliminary anticancer activity (93). Patients were given ABBV-176 once every three weeks with dose escalation based on level of exposure which was continually assessed. Drug-related toxicities were studied following each dose escalation to determine MTD. A group of 19 patients were enrolled, of which 11 had colorectal cancer, 6 had breast cancer and 2 had adrenocortical carcinoma. The patients were administered 2.7-109.36 μg/kg ABBV-176 (93). Dose-limiting toxicities occurred in four patients, which included two cases of thrombocytopenia, two cases of neutropenia, and one case of pancytopenia (93). The common adverse effects of ABBV-176 reported were thrombocytopenia, neutropenia, nausea, fatigue, increased aspartate aminotransferase, and pleural effusions. PRLR expression in tumors among these patients was varied, but no patient had an objective response. Unfortunately, there was considerable toxicity associated with ABBV-176 in this Phase 1 dose-escalation study. One caveat is that the study analysis relied on a small patient cohort with differential PRLR expression. This may be the reason why no response was observed. This study was terminated following administration of the drug to 19 patients. Therefore, further evaluation may be necessary with a larger cohort of patients with high PRLR expression.

##### 2.2.2.2 REGN2878-DM1

Another antibody-drug conjugate REGN2878-DM1 that is reported to target PRLR was developed by Kelly et al. to target PRLR positive breast cancer (31). This antibody-drug conjugate is composed of a high-affinity anti-PRLR IgG1 antibody conjugated to a cytotoxic maytansine derivative DM1, *via* a noncleavable Succinimidyl-4-(N-maleimidomethyl)cyclohexane-1-carboxylate linker. The antibody was generated in VelocImmune mice, which contain genes encoding human immunoglobulin heavy and kappa light chain variable regions. The mice were immunized with recombinant protein of the extracellular domain of human PRLR. Hybridomas were generated and joined to the human IgG1 constant region. REGN2878 was selected as the lead antibody after screening more than 300 antigen-binding clones. This antibody was further conjugated to DM1 *via* a non-cleavable SMCC (Succinimidyl 4-(N-maleimidomethyl)cyclohexane-1-carboxylate) linker and purified by size exclusion chromatography. The concentration of antibody-drug conjugate was confirmed by UV spectroscopy and MALDI-TOF mass spectrometry analysis. Both REGN2878 and REGN2878-DM1 were determined to have high-affinity binding to hPRLR with a K_D_ value of 1.05 and 1.24 nM respectively, and blocked prolactin binding to PRLR as measured by ELISA with an IC50 value of 5.0 and 4.4 nM, respectively (31). REGN2878-DM1 also inhibited prolactin-induced STAT5 activity in the HEK293/PRLR/STAT5-Luc reporter cell line, demonstrating inhibition of PRLR signaling. Moreover, REGN2878-DM1 treatment induced cell death in breast cancer cells with IC50 values between 0.06 nM and 0.97 nM (31). Proof of principle *in vivo* studies were performed using MCF7 and MCF7-PRLR over-expressing breast cancer xenograft mouse models in NCr nude mice. The xenografts were established and treated with a single dose, or thrice single-weekly doses of REGN2878-DM1 (5, 10, 15 mg/kg). Single dosing of 15 mg/kg significantly impaired tumor growth, which was also observed with 10 and 15 mg/kg repeated injections compared to control in both MCF7 and MCF7-PRLR overexpressing tumors (31). REGN2878-DM1 was further tested in breast cancer mouse xenografts of T47Dv11, which exhibit high levels of endogenous PRLR. As seen with previous studies, REGN2878-DM1 inhibited tumor growth in this model even at 2.5 and 5 mg/kg doses, while complete regression was observed with the highest 15 mg/kg dose (31). REGN2878-DM1 (2.5 mg/kg) was also test in combination with Fulvestrant (150 or 250 mg/kg), standard-of-care for ER+ breast cancer, showed greater inhibition of tumor growth in T47Dv11 xenografts in mice compared to monotherapy, suggesting synergistic or additive effects (31). Follow-up pharmacokinetic studies, in which 5 mg/kg of REGN2878-DM1 was delivered, resulted in serum levels above 16 μg/mL for at least 10 days, suggesting a long-lasting concentration sufficient to produce anti-tumor effects in the conducted mouse models (31). Collectively, these data suggest that the REGN2878-DM1 antibody-drug conjugate has potential to target PRLR and may have implications in the treatment of breast cancer with high expression of PRLR. Further early phase clinical trials will be required to assess toxicity, and preliminary efficacy in patients.

#### 2.2.3 Bispecific antibodies targeting PRLR

Bispecific antibody (BsAb) is a novel technology that contains two binding sites towards two different epitopes. This provides significant clinical advantages compared to monoclonal antibodies, due to an increased range of applications. Currently, more than 110 BsAbs are being evaluated in clinical trials (94), demonstrating the functionality, and excitement of this technology in targeting applications for human diseases and conditions. Two different BsAbs have been developed targeting PRLR.

##### 2.2.3.1 PRLR-DbsAb targeting CD3 and PRLR

Zhou et al. have recently developed a bispecific antibody, PRLR-DbsAb, that targets both PRLR and T-cell surface antigen, CD3 using the “Bispecific Antibody by Protein Trans-splicing” (BAPTS) system (95, 96). Briefly, Fragment A (CD3 antibody fusion protein) and Fragment B (PRLR antibody fusion protein) were expressed in CHO and 293E cell lines, respectively, and purified using protein L affinity chromatography. The authors identified that the PRLR-DbsAb-mediated cytotoxicity of immune effector cells is dependent on the ratio of effector to target cells; PRLR-DbsAb showed dramatic T-cell toxicity at the ratio of 5:1. Further, PRLR-DbsAb mediated cell killing of T47D (PRLR high) cells was 60% at a dose of 100 ng/ml at a ratio of 10:1 (97). It was shown that PRLR-DbsAb induced cytotoxicity *via* the synergistic effect of immune cell recruitment and not solely on the combined effect of PRLR and CD3 antibody. When T47D cells were treated with single targeting antibodies towards PRLR or CD3 alone, they produced less cytotoxic activity compared to PRLR-DbsAb. The EC50 values of PRLR-DbsAb against breast cancer cell lines MDA-MB-231, MCF-7, SKBR-3, and T47D were found to be 5.053, 1.78, 46.68, and 7.63 ng/ml, respectively (97). Mechanistically, PRLR-DbsAb was found to recruit T cells to PRLR expressing T47D breast cancer cells, which further induced cytotoxicity. Moreover, PRLR-DbsAb was found to activate T-cells *in vitro* as shown by increased CD69 levels in peripheral blood mononuclear cells (PBMCs) without target cells, while CD8^+^CD69^+^ T-cells had more activity than CD4^+^CD69^+^ T-cells when cultured with target cells (97). Rather, PRLR-DbsAb was found to activate CD4^+^CD69^+^ T-cells, while CD8^+^CD69^+^ T cell activation is dependent on combination with the target cells engagement. Moreover, cytokine release (IL10 and TNF-alpha) was significantly increased after PRLR-DbsAb treatment, supporting the T-cell activation mechanism (97). The *in vivo* activity of PRLR-DbsAb was evaluated in the NOD/SCID mice where T47D cells together with healthy human PBMCs were co-injected subcutaneously. PRLR-DbsAb was delivered once weekly at 0.33, 1, and 3 mg/kg intraperitoneally and compared with a 3 mg/kg PRLR monoclonal antibody (97). PRLR-DbsAb treatment significantly inhibited tumor growth at 0.33 mg/kg, which was comparable to PRLR monoclonal antibody alone. At higher doses of 3 mg/kg, PRLR-DbsAb substantially suppressed tumor growth, as both tumor volume and weight were impaired compared to control, and further increase survival of mice (97). Moreover, PRLR-DbsAb stimulated T-cell infiltration and expression of PD-L1 in these tumor tissues. Lastly, when PRLR-DbsAb was delivered in combination with a PD-1 antibody, anti-tumor activity was enhanced against MDA-MB-231 cells supporting the rationale of targeting PRLR with the novel BsAbs technology for PRLR-expressing breast cancers (97).

##### 2.2.3.2 Growth hormone receptor/Prolactin receptor BsAbs (H53)

As PRLR and growth hormone receptor (GHR) are closely involved in the incidence and development of breast cancer (98) which typically express PRLR, GHR, and GHR-PRLR heterodimers (99), the use of a combination PRLR and GHR antagonists may be a better strategy for breast cancer treatment. As such, Chen et al. have used a hybridoma technology to design a dual GHR-PRLR targeting antibody called H53 (100). Using competitive ELISA, receptor binding analysis, and immunofluorescence assays, the authors identified that H53 behaved like a typical anti-idiotypic antibody (Ab2β) (100). Further testing revealed that H53 treatment (0.05-1 μg/ml) inhibited not only the growth of CHO cells expressing PRLR and GHR but also PRLR-induced JAK2-STAT5 signaling (100). H53 also inhibited the PRL-induced phosphorylation of both STAT3 and STAT5, and AKT at a dose of 5-10 g/ml in T47D and MCF7 breast cancer cell lines, and further attenuated PRL-induced proliferation (100). Moreover, H53 also inhibited clonogenic potential, and migration that was accompanied by decreased expression of PRLR and GHR (100). The H53 BsAbs also displayed robust antitumor activity in proof-of-principle T47D and MCF-7 tumor xenografts models. When delivered at 15 and 30 mg/kg twice a week the expression p-STAT3/5 and p-AKT were downregulated in tumor tissue (100). H53-treated tumors also displayed a reduction in Ki67 that was accompanied by increased tunnel staining, indicating that H53 induced apoptosis in tumor cells. This preclinical study demonstrates the application of dual GHR/PRLR antibodies as a useful strategy for the treatment of breast cancer by impeding the PRLR signaling axis.

### 2.3 Small molecular inhibitors of PRLR

Extensive research has been conducted on developing antibody-based targeting and competitive antagonists of PRLR, but to date have unfortunately failed to produce sufficient anticancer activity in clinical trials. While these strategies have shown antagonization of PRLR in pre-clinical studies, poor bioavailability and stability can result in less durable responses, leading to tumor progression. While these technologies may still produce effective therapies, and certainly warrant further studies, an alternative solution to the noted clinical challenges may be resolved through the development of small molecule inhibitors. Advantages of small molecule inhibitors include oral delivery, low/no immunogenic properties, ability to cross the blood-brain barrier, easy to synthesize and optimize, and lower cost due to ease in manufacturing, transport, and storage compared to antibody based strategies (101). We have summarized below several studies focused on developing small molecule inhibitors for targeting PRLR, which have largely been conducted in the context of cancer.

#### 2.3.1 Small molecule inhibitors that target the ECD of PRLR

Borcherding et al. sought to identify a small molecular inhibitor targeting the extracellular domain (ECD) of PRLR (102). First, they performed *in silico* docking of a virtual library of 340,000 small molecules and evaluated their binding to the ECD of PRLR, of which 1000 compounds were predicted to affect PRL binding (102). Moreover, 50,000 diverse compounds were selected in addition to the predicted 1,000 compounds through virtual screening. For high-throughput screening, three sequential assays were performed on selected compounds. All three assays were designed under conditions where cells were incubated with PRL alone, compound alone, and PRL and compound in combination. Compounds that displayed significant cytotoxicity in the absence of PRL were eliminated. In the first assay, Nb2 cells, which are sensitive to PRL stimulation, were treated with selected compounds to determine the effect on proliferation at a concentration of 10 μM. The authors identified 120 potential compounds for further screening. In the second assay, a stably transfected PRLR cell line Ba/F3 was utilized for calculating IC50 values. Verification was performed in a third assay of T47D breast cancer cells stably transfected with luciferase reporter driven by a PRL-responsive promoter. Seven compounds were selected based on the IC50 values of 0.09 to 2.07 μM in the Ba/F3 assay. These were further analyzed for PRLR ECD binding using Microscale Thermophoresis (MST) technique. Three compounds, SMI-1, -6, and -7 bound to PRLR-ECD with K_D_ values of 1.26, 3.31, and 2.69 μM, respectively (102). Interestingly, SMI-1 was predicted by virtual screening and by molecular docking. Receptor binding was further confirmed by isothermal titration calorimetry. The ~40X ratio of antagonist/PRL binding affinities was found to be 1.26 μM vs. 29.9 nM (102). The incubation of SMI-1 and -6 at 1 μM concentration inhibited PRL-induced migration of MDA-MB-468 cells in the Boyden chamber transmigration assays. Moreover, both compounds also inhibited PRL-induced proliferation of Jurkat lymphocytes, as well as PRL-induced phosphorylation of JAK2 in Ba/F3 cells (102). SMI-6 was selected for further testing based on the absence of *in vitro* off-target toxicity. Further evaluation of SMI-6 identified that it inhibited PRL-induced phosphorylation of STAT5 in MDA-MB-468 cells without affecting the ability of growth hormone to phosphorylate STAT5 in PRLR deficient-T47D cells. To study the selectivity of SMI-6, the DiscoverX platform was used and tested against 168 G-protein-coupled receptors (GPCRs). In addition to PRLR, SMI-6 inhibited the serotonin receptors 2C, 2A, and hypocretin receptor 1 with IC50 values of 3.476, 2.395, and 6.712 μM, respectively. Moreover, SMI-6 was also tested against 468 kinases and failed to display inhibitory activity towards the tested kinases, including JAK2 (102). Subsequently, the authors evaluated the anti-proliferative activity in six breast cancer cell lines (BT474, MCF7, T47D, MDA-MB-231, ZR75-1, and MDA-MB-468). SMI-6 produced dose-dependent antiproliferative activity with IC50 values ranging from 0.29-1.68 μM (102). In non-malignant cells (fibroblasts, keratinocytes, and mammary epithelial cells) IC50 values were determined between 4.5-20.4 μM, suggesting low toxicity and a plausible therapeutic window (102). To confirm these findings, the authors assessed SMI-6 antitumor efficacy in proof-of-concept *in vivo* models utilizing athymic nude mice implanted orthotopically with control MDA-MB-468 cells or with Doxycycline regulated PRL producing cells (MDA-PRL). MDA-PRL produced larger tumors compared to control, while delivery of SMI-6 significantly inhibited tumor growth of MDA-PRL tumors. Moreover, SMI-6 did not show any apparent signs of toxicity or discomfort in mice. These data demonstrate that SMI-6 serves as a potent small molecular inhibitor targeting PRLR that may have implications for the treatment of breast cancer.

#### 2.3.2 Repurposing antipsychotic drugs for targeting the JAK-2 binding site of PRLR.

Several attempts were made to target the extracellular domain of PRLR using competitive antagonists, neutralizing antibodies, antibody-drug conjugates, and small molecule inhibitors but none have produced a clinically effective and acceptable antitumor response to date. In our own studies, we sought to identify novel targets involved in pancreatic ductal adenocarcinoma (PDAC) progression, and came across a pilot clinical trial that studied serum prolactin levels in women with different cancers (27). The authors observed 3-4 times greater prolactin levels in women with PDAC, which led us to investigate the role of PRL and PRLR in pancreatic cancer. In initial studies, we determined that PRLR is overexpressed in PDAC patient tissues by immunohistochemistry (25). The expression of PRLR in the normal pancreas was limited to the islet cells, while high cytoplasmic expression was observed in PDAC tissues. Moreover, we observed PRL released by PDAC tissues and cell lines using IHC and ELISA techniques, suggesting the role of both autocrine and paracrine PRL in PDAC progression. Furthermore, when we treated PDAC cells (MiaPaCa-2 and Panc-1) with PRL, it induced phosphorylation of canonical JAK2, STAT3, and ERK in a time- and dose-dependent manner, suggesting the functionality of the PRLR in PDAC cell lines (25). Interestingly, while PRL treatment failed to increase proliferation of MiaPaCa-2 and Panc-1 cells, we observed a significant increase in spheroid formation and migration (25). Furthermore, when we knocked down PRLR (PRLR KD) from PDAC cell lines (MiaPaCa-2 and mouse UKNC-6141) using CRISPR-Cas9 and shRNA approaches. PRLR KD resulted in significant inhibition of proliferation, colony formation, migration, and spheroid formation, suggesting that PRLR regulated multiple hallmarks of cancer progression (25). Moreover, when we treated PRLR knockdown cells with PRL, PRL failed to induce phosphorylation of JAK2, STAT3, and ERK suggesting the inhibition of PRL : PRLR regulated signaling pathways. In proof-of-concept studies, we injected PRLR knockdown UNKC-6141 cells in the pancreas of C57BL/6 mice to generate syngeneic orthotopic tumors. PRLR KD significantly impaired growth of orthotopic tumors compared to controls (25). These data suggested that PRLR affected PDAC progression and can be an attractive target for therapeutic interventions. These studies further demonstrate the feasibility and druggability of PRLR.

Due to limited the success achieved by targeting the PRLR ECD, we decided to approach targeting PRLR from a different perspective. In initial studies, we observed the presence of multiple isoforms of PRLR in PDAC cell lines. Structurally, all PRLR isoforms retain a conserved JAK2 binding domain. Following PRL binding to PRLR, JAK2 binding is the first downstream event that occurs in PRL-PRLR signaling. Hence, we sought to target the JAK2 binding domain of PRLR. We performed *in silico* virtual screening of small molecular inhibitors using a homology model of the intracellular domain (ICD) of PRLR, due to the lack of a published crystal structure for the ICD. We utilized I-TASSER software to predict inhibitors followed by virtual screening of small molecules using the IDOCK program. We selected two classes of compounds based on these predictions. We decided to use a fragment-based drug design approach to select commercially available small molecules. Since previous attempts achieved limited success in producing anticancer activity in clinical trials, we first screened these compounds for antiproliferative activity against PDAC cell lines. We found a single compound Penfluridol produced antiproliferative activity against MiaPaCa-2 and Panc-1 cells in a dose- and time-dependent manner, with an IC50 value of 3-4 μM concentration (25). Penfluridol is a first-generation antipsychotic drug used for the treatment of schizophrenia. We further performed multiple assays to study Penfluridol : PRLR binding and inhibition of PRL-induced signaling. First, we pretreated PDAC cells with Penfluridol at 4 μM concentration and subsequently stimulated with PRL. We determined that pretreatment of Penfluridol inhibited PRL-induced phosphorylation of STAT3 and ERK in both MiaPaCa-2 and Panc-1 cells (25). Moreover, we performed cell-based and cell-free drug-protein binding assays. Surface plasmon resonance and magnetic relaxometry using a peptide encoding the JAK-2 binding site of PRLR confirmed a dose-dependent response in Penfluridol binding (25). We further validated these results using cell-based binding assays. We performed a cellular thermal shift assay (CETSA), in which MiaPaCa-2 cells were treated with Penfluridol (5-20 μM) and subjected to a thermal gradient to assess PRLR denaturation in the presence or absence of drug. We observed that PRLR denatured at ~58°C, while denaturation occurred at 66°C in Penfluridol treated cells, suggesting that Penfluridol bound to PRLR and provided stabilization to thermal denaturation (25). These results were validated with the Drug Affinity Responsive Target Assay (DARTS). Similarly, Penfluridol provided stability to PRLR against pronase-induced proteolysis demonstrating Penfluridol binding. Collectively, these data confirmed that Penfluridol binds to PRLR.

We further tested the anticancer activity of Penfluridol in a variety of PDAC animal models. Penfluridol was delivered at 5 mg/kg intraperitoneally for 21 days in all models. We used UNKC6141 and KPC cell lines to generate orthotopic tumors in C57BL/6 mice. In a second model, we used Panc-1 cells to generate subcutaneous xenografts in Nude mice. In the third model, we generated a PDX in NSG mice. Penfluridol produced significant antitumor activity in all three animal models, and further induced LC3B and p62 mediated autophagy in PDAC cells as well as in orthotopic tumors (25). Our study is the first to target the JAK2 binding domain of PRLR. We demonstrate that Penfluridol binds to the PRLR ICD, and potently inhibits PRL : PRLR signaling that results in the inhibition of PDAC growth.

## 3 Summary and conclusions

It is becoming clear that PRLR mediated signaling plays a critical role in multiple human diseases and malignancies, and therefore is an attractive target for developing therapies. Since the early 1990s, researchers have attempted to generate PRLR antagonists and inhibitors with mixed success in pre-clinical and clinical applications. While none of these studies have resulted in FDA approval to date, they have provided a foundation for future discoveries that may yet be exploited. At the very least, our understanding of PRLR biology has expanded, and the studies to date have provided tools to interrogate this to greater depths.

First generation human PRL analogs exhibited weak agonistic activity towards PRLR despite numerous studies demonstrating antagonism, leading to reluctance for use in clinic (55). This contributed to the development of second generation analogs, such as Δ1–9-G129R-hPRL, which exhibits pure antagonism across multiple bioassays. Unfortunately, there remain challenges for clinical applications. Since these antagonists are small peptides (~23 kDa), these are quickly filtered by the kidneys, leading to suboptimal half-lives to maintain a potent and durable response. Nevertheless, the high selectivity of these analogs remains attractive with clinical prospects. With recent technological advancements, hormone based analogs may yet have therapeutic use. As shown in the recent studies by Yu et al., the fusion of second generation analogs with stabilizing proteins/peptides, such as albumin binding domain, can extend analog half-life substantially (71). As such, hormone analogs should not be discounted, and certainly further investigation is warranted to determine therapeutic implications.

Due to the clinical challenges innate to current hormone based analogs, and the significant advancement in antibody and protein engineering and recombinant DNA technology, antibody-based strategies have become of interest for antagonizing PRLR activity. Generally speaking, these strategies are attractive due to their success for the treatment of multiple human conditions, including cancer. Structurally, PRLR is an attractive target for antibody-based technologies due to the presence of a defined extracellular domain. Monoclonal antibodies and antibody-drug conjugates targeting PRLR have shown promising results in pre-clinical applications, antagonizing PRL induced signaling and cellular growth and migration (88–90, 92). Unfortunately, these have failed in Phase I clinical trials assessing toxicity and preliminary anti-tumor efficacy due to disease progression or development of dose-limiting adverse effects. This potentially could be improved by adjusting dosing frequency in therapies with minimal toxicities, though that is highly speculative. In such cases, antibody design is essential to preclinical and clinical success, and requires stringent study in regard to specificity, biodistribution, toxicity and efficacy. Moreover, antibody-drug conjugates are an exciting and novel technology. Though current designs have shown substantial toxicity in Phase I trials, there are significant advantages in concept design compared to monoclonal antibodies, combining the high specificity of antibodies and potency of cytotoxic drugs. Overall, there have been few antibody-based strategies that have been evaluated in clinical trials to date for targeting PRLR, largely due to the recency of technologic developments supporting their generation. As such, antibody based therapies may have significant potential for use in the future, though further study and designs are required to fully assess their prospective use.

The use of small molecule inhibitors may also provide alternatives to inhibiting PRLR signaling, and improving upon the challenges related to antibody and hormone based approaches. There are significant advantages to the use of small molecules; of particular note, oral delivery, ease of synthesis and optimization, and cost effectiveness make small molecule inhibitors a highly attractive approach (103). *In silico* screening tools combined with bioassays can provide a high-throughput screening pipeline for thousands of compounds. Prospective leads can then be validated for selectivity, and serve as scaffold platforms for additional analogs/derivatives to improve target binding, potency, bioavailability and stability.

To date, a handful of small inhibitors have been designed for targeting PRLR. SMI-6, developed by Borcherding et al., has been well characterized, demonstrating high selectivity for PRLR extracellular domain (102). Efficacy was also validated in both *in vitro* and *in vivo* models of breast cancer. Though SMI-6 is at the level of experimental investigation, the potential for therapeutic applications remains open, and certainly will be of interest to follow. In our own studies, we identified that Penfluridol, which has been approved for the treatment of schizophrenia, binds to PRLR at the JAK2 binding site within the intracellular domain (25). Penfluridol effectively inhibited PRLR signaling and maintained potent anti-tumor activity in multiple mouse models of PDAC (25). Penfluridol is a first generation antipsychotic with a long half-life, which may confer advantages in maintaining potent and durable responses in clinic. Unfortunately, Penfluridol is no longer licensed in the United States based on current FDA drug database information. Nevertheless, these studies demonstrate the feasibility of small molecule inhibitors targeting the intracellular domain of PRLR.

With the development of more accurate *in silico* screening tools, repurposing FDA approved drugs may serve as a means for rapid approvals for treating PRLR dependent conditions outside the original scope. FDA-approved drugs have been extensively screened for toxicity, safety, and pharmacokinetic and pharmacodynamic properties, and hence, may potentially decrease overall development timelines and costs for new applications. In this regard, It is important to address that the majority of PRLR targeting approaches have been designed against the extracellular domain, and it would be wise to expand these approaches to include the intracellular domain. Ultimately, the goal is to develop therapeutic strategies that can modulate PRLR signaling to promote positive clinical responses, either through agonistic or antagonistic mechanisms. For the purpose of studying PRLR biology, we already have numerous tools available that have been extensively characterized, and have been outlined in this review and **Table 1**, yet there remain challenges with the development of PRLR targeting therapeutics. As such, expanding our developmental strategies to include additional sites within PRLR may yield promising candidates for future clinical applications.

**Table 1:** Summary of PRLR inhibitors in preclinical and clinical stages of development.

Inhibitor	Class	Development Stage	Cancers tested	Effects	Reference
G129R-hPRL	hPRL protein analog	Preclinical	Ovarian, Breast	Inhibited cancer growth	(6, 21, 36–39, 41–46)
A1-9-G129R-hPRL	hPRL protein analog	Preclinical	Breast, Glioblastoma, Prostate	Inhibited cancer cell growth, chemoresistance	(47, 48, 50, 54–60)
ABD-PrIRA	Neutralizing antibody	Preclinical	Glioblastoma	Inhibited PRL induced signaling, extended serum half-life	(63)
LFA102	Neutralizing antibody	Clinical	Breast, Prostate	Inhibited cancer cell growth in preclinical studies, failed to inhibit tumor growth in clinical trials	(80–82)
F56	Neutralizing antibody	Preclinical	N/A	Inhibited PRL induced signaling	(83)
ABBV-176	Antibody-drug conjugate	Clinical	Breast, Prostate, Endometrial, Ovarian, Colorectal, Liver	Inhibited growth of cancer cells in preclinical studies, Clinical trial was stopped due to severe adverse toxicities	(84, 85)
REGN2878-DM1	Antibody-drug conjugate	Preclinical	Breast	Inhibited cancer growth	(22)
PRLR-DbsAb	Bispecific antibody	Preclinical	Breast	Activated T-cells, Inhibited cancer growth	(89)
H53	Bispecific antibody	Preclinical	Breast	Inhibited cancer growth, migration	(92)
SMI-6	Small molecule	Preclinical	Breast	Inhibited cancer growth, invasion	(94)
Penfluridol	Small molecule	Preclinical	Pancreas	Inhibited cancer growth, invasion, stemness	(18)

In summary, PRLR has become an attractive target for therapeutic development due to the broad expression of PRL and PRLR within biological tissues and human diseases. Hormone based approaches have yielded the development of specific antagonists, though their potential for clinical use is limited due to rapid filtration from blood and excretion. Antibody-based strategies have shown promise in preclinical applications, though they have failed in clinical trials due to toxicities and poor response. Nevertheless, there remains potential with antibody-based approaches due to the defined extracellular domain of PRLR. Similarly, the development of small molecule inhibitors has also shown potential in preclinical applications. The challenge now is to further assess lead candidates in clinical trials, as well as design new candidates with increased potency, with limited adverse toxicities.

## Author contributions

DS produced and edited the final manuscript. PD wrote the first draft of the manuscript. SA edited the final version of the manuscript. All authors contributed to the article and approved the submitted version.
